# Chronic Chagas cardiomyopathy: a review of the main pathogenic mechanisms and the efficacy of aetiological treatment following the BENznidazole Evaluation for Interrupting Trypanosomiasis (BENEFIT) trial

**DOI:** 10.1590/0074-02760160334

**Published:** 2017-02-16

**Authors:** Anis Rassi, José Antonio Marin, Anis Rassi

**Affiliations:** 1 Hospital do Coração Anis Rassi, Goiânia, GO, Brasil; 2 Universidade de São Paulo, Faculdade de Medicina de Ribeirão Preto, Departamento de Clínica Médica, Divisão de Cardiologia, Ribeirão Preto, SP, Brasil

**Keywords:** chronic Chagas cardiomyopathy, Chagas heart disease, pathogenesis, aetiological treatment, Benznidazole, BENEFIT trial

## Abstract

Chagas cardiomyopathy is the most frequent and most severe manifestation of chronic Chagas disease, and is one of the leading causes of morbidity and death in Latin America. Although the pathogenesis of Chagas cardiomyopathy is incompletely understood, it may involve several mechanisms, including parasite-dependent myocardial damage, immune-mediated myocardial injury (induced by the parasite itself and by self-antigens), and microvascular and neurogenic disturbances. In the past three decades, a consensus has emerged that parasite persistence is crucial to the development and progression of Chagas cardiomyopathy. In this context, antiparasitic treatment in the chronic phase of Chagas disease could prevent complications related to the disease. However, according to the results of the BENEFIT trial, benznidazole seems to have no benefit for arresting disease progression in patients with chronic Chagas cardiomyopathy. In this review, we give an update on the main pathogenic mechanisms of Chagas disease, and re-examine and discuss the results of the BENEFIT trial, together with its limitations and implications.

Chagas heart disease has two phases, acute and chronic, usually separated by several decades. Most patients in the chronic phase have the indeterminate form of the disease for life, which is characterised by absence of signs and symptoms of disease, a normal 12-lead electrocardiogram, and normal radiological examination of chest, oesophagus, and colon. Even in these patients an array of subtle anatomic and functional abnormalities has been shown with the use of more elaborate diagnostic tests, demonstrating that, similar to other diseases, Chagas disease manifests in humans with an ample spectrum of severity (Mady et al. 1984, Décourt et al. 1985). Despite the presence of such minor abnormalities, whose prognostic meanings are uncertain and are detected only with more sophisticated methods, it is important to emphasize that as long as the patients remain with the indeterminate form of the disease their prognosis is excellent (Dias 1989, de Oliveira Jr 1990). However, a most intriguing question regarding the pathogenesis of chronic Chagas disease is which genetic or acquired factors are decisive to determine in a specific patient the installation and progression of the cardiomyopathy (Marin-Neto et al. 2010, Rassi Jr et al. 2010).

*Pathogenic alterations in acute Chagas disease* - Organ damage during the acute phase occurs as a result of intense parasitaemia and tissue parasitism, with superimposed immune-inflammatory response to the parasite. Although any organ can harbour the parasites, experimental *Trypanosoma cruzi* infection has a typical predilection for the muscle system in the heart, oesophagus and colon, and for the central nervous system (Okumura et al. 1960, Teixeira et al. 1975, Andrade et al. 1994). An in vitro model using human cell lines in culture to observe *T. cruzi* passage through the vascular barrier showed that there is usually no disruption of the endothelial monolayer, as the parasite uses a special transmigration process that is facilitated by bradykinin and CCL2 chemokine (Coates et al. 2013). These interesting findings still await for a demonstration that such phenomenon also occurs in vivo. Conventional histopathology shows prominent inflammatory changes in the vicinity of ruptured infected cells in tissues from both right and left cardiac chambers (Laranja et al. 1956, Kumar et al. 1969). Myocarditis is intense and diffuse with myocyte necrosis, interstitial oedema, vasculitis and capillary dilation, and mononuclear and polymorphonuclear infiltration (Okumura et al. 1960, Kumar et al. 1969, Teixeira et al. 1975, Andrade et al. 1994). The immunological reaction is thought to control the active parasite multiplication through various host innate mechanisms that play a role in detecting and controlling parasite tissue invasion - a powerful reaction involving CD4+ and CD8+ T-cells and B-cells activation that induces direct antitrypanosoma cytotoxicity, cytokine secretion and production of specific antibodies against the parasite (Tarleton et al. 1992).

The neuronal depopulation of the Meissner and Auerbach plexuses that occurs in oesophageal and colon tissues during the acute phase are a key factor in the pathogenesis of megaoesophagus and megacolon in the chronic phase (Köberle 1968, Meneghelli 1985). Direct damage of smooth muscle may also be a contributory factor, but this hypothesis has not been adequately explored in humans and animal models of Chagas disease.

*Pathogenesis of chronic Chagas cardiomyopathy* - The pathogenic mechanisms responsible for cardiac lesions developing during the chronic phase of Chagas disease are not completely understood, but four mechanisms are believed to contribute: neurogenic disturbances, microvascular derangements, parasite-dependent damage, and immune-mediated tissue injury (Marin-Neto et al. 2007).

The first two mechanisms probably play only an ancillary role in the development of the cardiac lesions and clinical complications observed in patients with chronic Chagas cardiomyopathy, and will not be discussed here (Marin-Neto et al. 2007, 2010, 2013). By contrast, most investigators now believe that parasite persistence is a critical factor in causing inflammation and in initiating and progressing chronic myocarditis. This previously neglected concept in turn rescues the notion that chronic Chagas disease is indeed an infectious entity, in which the parasite is not completely eliminated, despite the multiple and proteiform reactions developed by the host systems against it.

*Parasite-dependent inflammation and myocardial damage* - Chronic Chagas cardiomyopathy is an acquired cardiomyopathy characterised by sparse inflammatory infiltrates, minimal parasitaemia, low-grade tissue parasitism, and intense and extensive reparative and reactive fibrosis (Rossi 1991). Classical histological techniques usually cannot detect the parasite, but immunohistochemical and polymerase chain reaction (PCR) based methods have identified *T. cruzi* antigens in inflammatory foci in biopsy and autopsy materials from patients with chronic Chagas disease (Higuchi et al. 1993, Bellotti et al. 1996). Thus, a consensus is now emerging that parasite persistence is directly and causally related to cell death and parasite-driven immune responses, which play a pivotal role in the development of chronic Chagas cardiomyopathy (Tarleton 2003a, Kierszenbaum 2007, Bonney & Engman 2008).

Additional evidence to support this concept has been found in other studies in animal models of *T. cruzi* infection and in humans with Chagas disease: (1) tissue parasite load clearly correlates with the intensity of inflammation in animal models of *T. cruzi* infection (Zhang & Tarleton 1999); (2) reinfection or continued exposure to infection by permanently residing in areas of active transmission leads to an increase of both the parasite load and disease severity in animal models and in infected humans (Bustamante et al. 2002, Storino et al. 2002); (3) trypanocidal treatment with benznidazole, nifurtimox, or fexinidazole does not eradicate the parasite, but the reduced parasite burden does attenuate the myocarditis in animal models (Andrade et al. 1991, Garcia et al. 2005, Bahia et al. 2012); (4) *T. cruzi* genetic material has been consistently detected in cardiac specimens from patients with chronic Chagas cardiomyopathy, but not in cardiac specimens from seropositive patients who died without clinical signs of cardiac disease (Jones et al. 1992); 5) *T. cruzi* DNA was detectable by PCR methods in the peripheral blood of 86% of patients with well-defined chronic Chagas cardiomyopathy (Salomone et al. 2000).

Marked phenotype and genotype diversity occurs among the six classes of *T. cruzi* strains (Zingales et al. 2012). This may be a cause for the remarkable differences in the pathological and clinical manifestations of Chagas disease in various geographical regions – e.g., virtual absence of gastrointestinal disease, or discrepancies in the incidence of sudden death (Zingales et al. 2014). *T. cruzi* genetic diversity may also be responsible for the inconsistent response to several trypanocidal agents in animal and clinical studies (Rassi Jr et al. 2009).

*Immune-mediated tissue injury* - Immune-mediated cardiac injury is caused by the infiltration of mononuclear cells and release of damaging cytokines, which play a decisive role in the development of chronic Chagas cardiomyopathy, according to animal and clinical studies (Marin-Neto et al. 2015a). These mechanisms are most probably triggered by persistence of the parasite in the tissue, but autoimmunity mechanisms – involving polyclonal activation, molecular self-mimicry by parasite antigens, or cryptic epitopes shared by the host and parasites – have also been reported in animal models and humans with Chagas disease, and are thought to contribute to or aggravate myocardial damage (Minoprio 2001, Cunha-Neto et al. 2006, Teixeira et al. 2011a).

However, the autoimmunity hypothesis – that polyclonal activation or molecular mimicry is directly involved in the pathogenesis of myocardial lesions ascribed to *T. cruzi* infection – remains controversial and difficult to validate (Tarleton & Zhang 1999, Tarleton 2003b). Anti-self responses are described in *T. cruzi* infection, but there is no direct and definitive evidence that the immune reactions against the mimicked auto-antigens are actually pathogenic, as the anti-self antibodies in animal models and humans with chronic Chagas disease are heterophilic and have a poor correlation with development of heart lesions (Tarleton 2003b). In summary, the role, relative contribution and clinical relevance of autoimmunity in triggering myocardial degeneration in the chronic phase of Chagas disease remains to be determined (Marin-Neto et al. 2015a).

An additional mechanism for the autoimmune response in the absence of parasites was suggested by the observation that mitochondrial DNA from *T. cruzi* can be inserted into the genome of a chicken model in which the parasitic infection was induced at the egg stage, but parasite persistence was precluded (Teixeira et al. 2011b).

Immune-mediated pathology of chronic Chagas cardiomyopathy is rather complex, probably involving several interactive factors. This complexity is illustrated by the paradox observation that natural or iatrogenic immunosuppressive conditions usually exacerbate *T. cruzi* parasitaemia and aggravate the inflammatory process (Rassi et al. 1997, Sartori et al. 2007). This epitomises the double-edged-sword type of host immune response to the parasite, because the inflammatory lesions found in the myocardium of patients and animals chronically infected with the *T. cruzi* are typically composed of macrophages and a predominance of CD8+ over CD4+ Th1 cells (Dutra & Gollob 2008). The pathogenic picture is further compounded by the enhanced expression of genes responsible for an increased release of several pro-inflammatory cytokines and chemokines, especially INF-g and TGF-a (Ferreira et al. 2014). Also, other investigators have reported a reduced production of regulatory T-cells and their related cytokines, IL-10 and IL-17 (Guedes et al. 2012, Nogueira et al. 2014). These findings are consistent with an immunological imbalance related to up-regulation of Th-1-cells, and deficient suppressor activity of regulatory T-cells that otherwise would act to control myocardial inflammation.

There is now ample evidence that the immunopathology reactions in chronic Chagas cardiomyopathy are dependent on genetic polymorphisms of the host, which modulate the expression of immune inhibitory molecules and potentially alter the equilibrium between host and parasite (Marin-Neto et al. 2015a). Thus, alleles, genotypes and haplotypes associated with enhanced expression of the regulatory CTLA-4 system predominate in patients with the indeterminate form of Chagas disease, probably averting the development of cardiomyopathy (Dias et al. 2013). By contrast, in a genetic and proteomic study of infected patients with chronic Chagas cardiomyopathy, polymorphism in the alfa cardiac actin-1 gene (ACTC-1) was associated with an increased tendency to maintain a pro-inflammatory status, possibly by modulating transcription factor binding to ACTC-1 promoter regions (Frade et al. 2013). These results and others from several investigators confirm previous evidence of familial aggregation of cases with chronic Chagas cardiomyopathy, and hint that only around a third of infected patients develop the clinical complications of the disease because of a genetic component that confers susceptibility after infection (Cunha-Neto & Chevillard 2014).

Contrary to previous reports, more recent evidence suggested that spontaneous eradication of the parasite may be achieved by the effective action of the host immune system in the murine model of *T. cruzi* infection (Tarleton 2013), and there have been several anecdotal reports of spontaneous cure of *T. cruzi* infection in humans (Francolino et al. 2003, Dias et al. 2008). A recent hypothesis was developed to explain why the immune system may not always be capable of sterilising these infected animals or the human host: instead of an inherently deficient immune response, it is possible that the parasite could escape the cytotoxic CD8+ cells due to its ability to remain unnoticed within myocardial and other harbouring structural cells (Álvarez et al. 2014).

Finally, a pathophysiological link between impaired parasympathetic control, a derangement described in early phases of human Chagas disease, and abnormal neuroimmunomodulatory regulation has been recently suggested in a murine model of chronic *T. cruzi* infection (Cuba et al. 2014). These findings were based on the effect of pharmacological cholinergic stimulation using pyridostigmine, a cholinesterase inhibitor, which reduced myocardial inflammation, fibrosis, hypertrophy, and serum levels of IFN-g, but did not change IL-10 levels. Cuba et al. (2014) thought that the autonomic dysregulation caused by *T. cruzi* infection could abolish the normal neuroimmunomodulatory anti-inflammatory role that is normally played by the parasympathetic nervous system.

*Evidence for the benefit of aetiological treatment in patients with chronic Chagas disease* - Until the BENznidazole Evaluation for Interrupting Trypanosomiasis (BENEFIT) trial (Marin-Neto et al. 2008, Morillo et al. 2015), evidence to support the fact that treatment with effective trypanocidal drugs (nifurtimox or benznidazole) can positively affect the progression of disease in asymptomatic patients with *T. cruzi* infection or in patients with preexisting cardiac disease was scanty and based mostly on observational studies and a few small randomised trials. Table I shows the systematic reviews and meta-analyses looking at this, and a systematic review of people with *T. cruzi* infection in the USA also included a critical appraisal of trypanocidal treatment (Bern et al. 2007).

**TABLE I:** Reviews of the effect of aetiological treatment in patients with chronic Chagas disease

	Number of patients	Stage of Chagas disease	Number and type of studies	Follow up	Outcomes
Villar et al. (2002)	756	Chronic phase (asymptomatic)	Five small randomised trials	1-4 years	None of the studies assessed clinically relevant hard outcomes, and two tested ineffective drugs (itraconazole and allopurinol) versus placebo, instead of benznidazole or nifurtimox. Overall, parasite-related outcomes were significantly (statistically) improved, including the seroconversion rate (OR 10.91, 95% CI 6.07-19.58), xenodiagnoses conversion rate (OR 5.37, 95% CI 3.34-8.64), and standardised mean reduction of antibody titres (OR 0.54, 95% CI 0.31-0.84).
Reyes and Vallejo (2005)	714^*a*^	Chronic phase (asymptomatic and symptomatic)	One small randomised trial, and six uncontrolled or non-randomised studies	1-23 years^*a*^	Overall results were insufficient to draw any conclusions.
Pérez-Molina et al. (2009)	1924	Chronic phase (asymptomatic and symptomatic)	Three randomised trials, and six observational studies	1-24 years^*a*^	Available information comparing benznidazole versus placebo or no treatment showed that children treated with benznidazole had a better tolerance and better parasite-related responses than adults did. More importantly, overall patients treated with benznidazole had a significantly lower risk of clinical events than those treated with placebo (OR 0.29, 95% CI 0.16-0.53).
Villar et al. (2014)	4229	Chronic phase (asymptomatic)	Six randomised trials, and seven observational studies	At least 4 years	Ten studies tested nifurtimox or benznidazole versus placebo and showed potentially important, but imprecise and inconsistent reductions in progression of chronic Chagas cardiomyopathy (four studies, 106 events, OR 0.74, 95% CI 0.32-1.73, I^2^ = 66%) and mortality (six studies, 99 events, OR 0.55, 95% CI 0.26-1.14, I^2^ = 48%).

*a*: calculated from the original studies included in the reviews; OR = odds ratio.

Several obstacles have hindered gaining a better understanding, including (Andrade et al. 2011): the misconception that the main pathogenic mechanism of chronic Chagas disease was autoimmunity, not parasite persistence; the fact that no ideal trypanocidal drug has been developed since nifurtimox and benznidazole were introduced 40 years ago (so far these are the only clinically approved drugs that are proven to be active against both the circulating and the tissue-nested parasites); the wrong belief that side-effects related to these drugs were too frequent and serious to be tolerated by most patients; and doctors’ natural reluctance to use an aetiological treatment in patients with already manifest cardiomyopathy, because they believed it would be too late for any benefit.

*Development of the protocol for the BENEFIT trial* - Two of us (ARJ and AR) wrote the original draft protocol for a randomised, double-blind, placebo-controlled trial to assess whether trypanocidal therapy with benznidazole for 60 days reduces mortality and major cardiovascular events in patients with established chronic Chagas cardiomyopathy (Marin-Neto et al. 2008). We suggested the research should be multicentre and international, and mean follow-up should last for 5 years. This proposed trial followed a visit to Brazil in 2002 by Dr Salim Yusuf from the Population Health Research Institute, Hamilton Health Sciences and McMaster University (Hamilton, ON, Canada), who was keen to sponsor a collaborative research programme.

Table II sets out some features of the original protocol and shows that the final protocol changed in several key ways following input from the BENEFIT steering committee.

**TABLE II:** Key features of the original protocol and differences in the final protocol, which were agreed by most members of the BENznidazole Evaluation for Interrupting Trypanosomiasis (BENEFIT) steering committee

	Original protocol	Final BENEFIT protocol
Eligibility criteria		
Age	18-50 years	18-75 years
Evidence of chronic Chagas cardiomyopathy	Positive serological tests for *T. cruzi*, and electrocardiographic or echocardiographic alterations, or both, that were characteristic of chronic Chagas cardiomyopathy	Same criteria
NYHA functional class	I or II, exclude those with congestive heart failure (NYHA III or IV)	I, II or III
Living in conditions that predispose to *Trypanosoma cruzi* infection	Exclude	Include
Previous resuscitation following cardiac arrest	Exclude	Include
Previous sustained ventricular tachycardia	Exclude	Include
Previous insertion of a pacemaker or cardiac defibrillator	Exclude	Include
Previous admission to hospital for heart failure	Exclude	Include
Previous thromboembolic event	Exclude	Include
Endpoints		
Primary	Composite of time to cardiovascular death, resuscitated cardiac arrest, sustained ventricular tachycardia, insertion of a pacemaker or cardiac defibrillator, admission to hospital for heart failure, and development of thromboembolic events.	Composite of time to death, resuscitated cardiac arrest, sustained ventricular tachycardia, insertion of a pacemaker or cardiac defibrillator, cardiac transplantation, and development of new heart failure, stroke, or systemic or pulmonary thromboembolic events.
Secondary	Composite of electrocardiographic and echocardiographic changes, as markers of disease progression (surrogate endpoints), throughout the study period. Eventual differences in outcomes between individual countries	Secondary outcomes also included the response to treatment on the basis of results on PCR assay.
Statistical analysis		
Sample size	3000 patients (1500 per group) needed to detect a 20% reduction in the relative risk of the primary endpoint in the benznidazole group with 90% power, assuming a 5-year event rate of 30% in the placebo group (at a two-sided α of 0.05). Expect to lose 20% of patients from non-compliance or during follow-up	3000 patients (1500 per group) needed to detect a 26% reduction in the relative risk of the primary endpoint in the benznidazole group with 90% power, assuming a yearly event rate of 8% in the placebo group and 4-6 years of follow-up (at two-sided α of 0.05). Expect to lose 17% of patients from non-compliance and 3% during follow-up.

NYHA: New York Heart Association.

We recommended excluding patients with advanced heart disease or who already manifested a clinical condition that was a component of the composite primary endpoint of the study, but the final protocol opted to substantially broaden the eligibility criteria. We also disagreed with the decision to include older patients (up to 75 years), and patients who were susceptible to reinfection.

We derived a projected event rate of 30% in the placebo group from the results of the doctoral thesis of one of us (Rassi Jr 2003). In this longitudinal study of 424 Brazilian patients with chronic Chagas cardiomyopathy in the 1980s, the combined event rate was 34% after 5 years of follow up. The mean age of the cohort was 47 years (SD 11.0); patients older than 70 years were excluded and only five patients were aged 65-70 years.

We considered a 20% relative risk reduction – from an event rate of 30% in the placebo group to 24% in the benznidazole group – as the minimal clinically important reduction in effect size, worthy to justify a change in the patient’s management. It represents an absolute risk reduction of 6%, which translates into a number needed to treat of 17; that is, we would have to treat 17 patients with benznidazole for 60 days to prevent one major cardiovascular event, after 5 years of follow-up. We also chose a 20% relative risk reduction because it was considered feasible (although not easy) to achieve. 10-20% of patients cannot tolerate taking benznidazole, efficacy of benznidazole in eradicating *T. cruzi* is limited in the patients we had deemed eligible (less than 50%, at best), and, even if benznidazole eliminates the parasite, the mechanism of disease progression may not be exclusively parasite-related. Thus, proper selection of patients for whom trypanocidal therapy has at least some plausibility to work is of paramount importance in a trial of a drug and a disease with such peculiarities. Although extending the eligibility criteria helps with recruiting patients and generalising the results, the inclusion of patients who are not likely to respond to benznidazole could dilute identifying an eventual beneficial effect.

Very importantly, we recommended that the study should focus on the assessment of hard clinical endpoints, not parasite-related outcomes, such as clearance of parasitaemia or disappearance of antibodies (negative seroconversion). The results of conventional serological assays remain positive for years or even decades after successful therapy (Rassi & Luquetti 2005), and the negative results of PCR assays after treatment are not reliable markers of cure (Britto et al. 2001, d, de Lana & Martins-Filho 2015). A negative PCR result does not necessarily rule out infection; it indicates only the absence of circulating *T. cruzi* DNA in the blood sample being drawn for testing. Therefore, these tests are neither useful nor practical to assess the outcome of antitrypanosomal therapy in patients with chronic Chagas cardiomyopathy, and we disagreed with the BENEFIT steering committee’s decision to further assess the effect of benznidazole on parasite clearance using PCR and whether this translates to improved clinical outcomes. Instead, in the original protocol, a pilot study involving PCR investigation, designed exclusively to test if aetiological treatment significantly reduces parasite burden in the first 600 patients recruited to the main trial was proposed (Marin-Neto et al. 2008).

*Principal results of the BENEFIT trial* - BENEFIT is the largest randomised trial on Chagas disease (Morillo et al. 2015) and the investigators are commended for achieving such a large trial in a disease where recruitment of patients is notoriously difficult. It involved 2854 patients with chronic Chagas cardiomyopathy at 49 centres in five Latin American countries: 1358 patients in Brazil, 559 in Argentina, 502 in Colombia, 357 in Bolivia, and 78 in El Salvador. Patients were assigned to receive benznidazole or placebo (5 mg per kg of bodyweight per day) for 40-80 days, and followed up for a mean of 5.4 years (Morillo et al. 2015).

Benznidazole had no significant effect on the primary endpoint that was the first event of any of the components of the composite outcome of death, resuscitated cardiac arrest, sustained ventricular tachycardia, insertion of a pacemaker or implantable cardioverter-defibrillator, cardiac transplantation, new heart failure, stroke, or other thromboembolic event. 27.5% of patients in the benznidazole group versus 29.1% in the placebo group reached the primary endpoint, corresponding to a hazard ratio (HR) of 0.93 (95% CI 0.81-1.07; p = 0.31) (Morillo et al. 2015).

PCR was used to detect blood parasites in 1896 patients at baseline, in 1618 patients at the end of treatment, in 1530 patients at two years, and in 1487 patients at the final follow-up visit (Morillo et al. 2015). PCR had low sensitivity to blood parasite DNA – it was detected in around 60% of patients at baseline (59.5% of patients in the benznidazole group vs 61.7% in the placebo group tested positive). Additionally, the test was not done in the same patients at each scheduled follow-up visit after treatment (number of patients who had all tests done was not informed).

For patients who tested positive for blood parasites at baseline, treatment converted the PCR result to negative in 66.2% of patients in the benznidazole group versus 33.5% in the placebo group by the end of the treatment period, in 55.4% versus 35.3% at two years of follow-up, and in 46.7% versus 33.1% at five years or more of follow-up (p < 0.001 for all comparisons) (Morillo et al. 2015).

The proportion of patients whose PCR result converted to negative varied between countries. For example, at the end of treatment, PCR conversion was highest in Brazil, with 86.3% of patients in the benznidazole group versus 24.3% in the placebo group [odds ratio (OR) 7.20, 95% CI 4.53-11.4], and it was lowest in Colombia and El Salvador, with 45.6% in the benznidazole group versus 43.9% in the placebo group (OR 1.15, 95% CI 0.81-1.62). In Argentina and Bolivia, 73.0% of patients in the benznidazole group versus 28.6% in the placebo group converted to negative (OR 3.32, 95% CI, 2.43-4.54) (Morillo et al. 2015).

Although 23.9% of patients in the benznidazole group had transiently interrupted treatment because of adverse events (vs 9.5% for placebo, p < 0.001), only 13.4% discontinued treatment permanently (vs 3.6% for placebo, p < 0.001), which is lower than previously reported with benznidazole. Notably, only 14 patients (0.5%) were lost to follow-up by the end of the study (Morillo et al. 2015).

*Limitations of the findings* - Several features of the BENEFIT trial raise some concerns and merit further discussion before concluding that benznidazole has no role in the treatment of patients with established chronic Chagas cardiomyopathy. We exhaustively addressed all these concerns during the meetings of the BENEFIT steering committee.

Despite the neutral overall result, all components of the primary endpoint were, albeit not attaining statistical significance, less frequent in the benznidazole group than in the placebo group (Table III). Table III also shows that patients receiving benznidazole had significantly fewer admissions to hospital for cardiovascular causes than those receiving placebo. It is possible that the results of BENEFIT could become positive if admission to hospital was included in the composite endpoint, as we suggested in the original protocol (Table II). Keeping patients out of hospital is a major goal in the treatment of patients with chronic Chagas cardiomyopathy and, even as a post-hoc analysis, this finding may be informative.

**TABLE III:** Proportion of patients who reached the endpoints of the BENznidazole Evaluation for Interrupting Trypanosomiasis (BENEFIT) trial

	Benznidazole (n = 1431)		Placebo (n = 1423)		Unadjusted hazard ratio (95% CI)	p value
	
	Patients with event	Event rate per year^*a*^	Patients with event	Event rate per year^*a*^		
Primary endpoint	394 (27.5%)	5.1%	414 (29.1%)	5.4%	0.93 (0.81-1.07)	0.31
Components of the primary endpoint						
Death	246 (17.2%)	3.2%	257 (18.1%)	3.4%	0.95 (0.79-1.13)	-
Resuscitated cardiac arrest	10 (0.7%)	-	17 (1.2%)	-	0.58 (0.27-1.28)	-
Sustained ventricular tachycardia	33 (2.3%)	-	41 (2.9%)	-	0.80 (0.50-1.26)	-
Pacemaker or cardiac defibrillator	109 (7.6%)	-	125 (8.8%)	-	0.86 (0.66-1.11)	-
Cardiac transplantation	3 (0.2%)	-	9 (0.6%)	-	0.33 (0.09-1.22)	-
New or worsening heart failure	109 (7.6%)	-	122 (8.6%)	-	0.88 (0.68-1.14)	-
Thromboembolic event or TIA	54 (3.8%)	-	61 (4.3%)	-	0.88 (0.61-1.26)	-
Exploratory outcome						
Admission to hospital for cardiovascular causes	242 (16.9%)	-	286 (20.1%)	-	0.83 (0.70-0.98)	0.03

Data are number (%) unless otherwise stated. TIA: transient ischaemic attack; *a*: the event rate per year was calculated dividing the percentage of patients with an event by the mean follow-up time for the overall population (5.38 years). Event rates are given for the primary endpoint and death only. Adapted from Morillo et al. (2015).

Although composite outcomes are frequently adopted as the primary endpoint in clinical trials, analysing time to first event is suboptimum for a chronic illness characterised by a relatively long period before death, during which multiple non-fatal events may occur, often leading to hospital admission (Rogers et al. 2014). For a trial of chronic Chagas cardiomyopathy, an analysis using statistical methods that take into account recurrent events could show a larger treatment benefit than the conventional analysis of time to first event, and is worthy of further investigation.

Most investigators of the BENEFIT trial believed that the baseline characteristics of the two study groups were well balanced at the time of randomisation. However, five of six factors that are clearly recognised as strong predictors of a poor outcome (Rassi Jr et al. 2006) were more common in the benznidazole group (Table IV). Unfortunately, no adjustment for these specific variables was reported.

**TABLE IV:** Imbalances at baseline of the BENznidazole Evaluation for Interrupting Trypanosomiasis (BENEFIT) trial in the six prognostic factors in the Rassi score, which is used to assess risk of death in chronic Chagas cardiomyopathy

	Benznidazole (n = 1431)	Placebo (n = 1423)
Male sex	50.7%	47.9%
Low QRS voltage on electrocardiography	13.3%	12.1%
Complex ventricular arrhythmia	15.4%	13.3%
Wall motion abnormality^*a*^	38.3%	37.6%
Evidence of cardiomegaly on radiography	NA	NA
NYHA functional class III	2.7%	2.3%

*a*: echocardiography was done less than 1 year before randomization; NA: not available; NYHA: New York Heart Association. Adapted from Morillo et al. (2015).

*Was BENEFIT an underpowered clinical trial?* - The BENEFIT investigators estimated that 3000 patients (1500 in each group) were needed to detect a 26% reduction in the relative risk of the primary endpoint with benznidazole compared with placebo, or vice versa, with 90% power (Table II). However, the event rate in the placebo group was lower than expected (5.4% instead of 8% per year), so the BENEFIT trial was underpowered to detect significant differences in cardiovascular events between the two groups.

Table V shows that even if the event rate in the placebo group had been 6% per year (as predicted in the original protocol estimate), the relative risk would have had to reduce by at least 28.8%, according to the final BENEFIT protocol calculations, to maintain a power of 90% with the same sample size and get a p value of less than 0.05. We speculate that such a large reduction in the relative risk of events is highly unlikely to occur when patients with established chronic Chagas cardiomyopathy are treated with benznidazole, and planning for a modest improvement in outcomes would probably be a more realistic strategy. Reductions of 15-25% in relative risk and 5% in absolute risk are sufficiently worthwhile to change clinical practice in this population, and align with the estimates we made in the original protocol (Table II).

**TABLE V:** Expected relative risk reduction depending on the sample size and proportion of patients in the placebo group who reached the primary endpoint per year in the BENznidazole Evaluation for Interrupting Trypanosomiasis (BENEFIT) trial

Annual event rate (placebo group)	Relative risk reduction depending on sample size		

	2000 patients (1000 per group)	2500 patients (1250 per group)	3000 patients (1500 per group)
6%	34.9%	31.3%	28.8%
7%	32.7%	29.6%	27.0%
8%	30.6%	27.6%	25.6%
9%	29.0%	26.5%	24.1%
10%	28.0%	25.4%	23.0%

Calculations assume a two-sided α of 0.05, and a power of 90%. Adapted from Marin-Neto et al. (2008).

*Efficacy of benznidazole between geographical regions* - Response to treatment did not differ significantly between geographical regions, according to a post-hoc subgroup analysis of the BENEFIT trial (Table VI). This conclusion was based on a statistical test of interaction, which assessed whether the variation in hazard ratios across regions could plausibly have arisen by chance.

**TABLE VI:** Proportion of patients who reached the primary endpoint in the countries participating in the BENznidazole Evaluation for Interrupting Trypanosomiasis (BENEFIT) trial

	Benznidazole group		Placebo group		Unadjusted hazard ratio (95% CI)	p value for interaction
	
	Proportion of patients, with event	Event rate per year^a^	Proportion of patients, with event	Event rate per year^*a*^		
Brazil (n = 1358)	33.2%	6.0%	37.6%	6.8%	0.85 (0.71-1.02)	0.16
Colombia and El Salvador (n = 580)	24.1%	4.7%	25.6%	5.0%	0.92 (0.66-1.27)	
Argentina and Bolivia (n = 916)	21.4%	4.0%	18.5%	3.5%	1.18 (0.88-1.58)	

*a*: the yearly event rate was calculated by dividing the proportion of patients with an event by the mean follow-up time: 5.54 years for Brazil, 5.10 years for Colombia and El Salvador, and 5.31 years for Argentina and Bolivia. Adapted from Morillo et al. (2015).

However, subgroup analysis by geographical region is particularly challenging because countries can be grouped in many different ways, each yielding a different strength of evidence for geographical heterogeneity (Pocock et al. 2013). Additionally, the overall benefit of a new treatment may not apply equally to all eligible patients; this is particularly true in Chagas disease because genetic subtypes of *T. cruzi* vary across geographical regions, and diversity in parasite genotyping may affect response to benznidazole (Filardi & Brener 1987, Neal & van Bueren 1988, Yun et al. 2009).

In the BENEFIT trial, no genotype analyses were done. The grouping of geographical regions was not predefined before the study started, and followed the assumption that the strain of *T. cruzi* varies between countries, based on available studies (Zingales et al. 2012, 2014): Colombia and El Salvador (where strain TcI is most prevalent), Brazil (where strain TcII is most prevalent), and Argentina and Bolivia (where strains TcV and TcVI are most prevalent). If all five countries are instead compared separately, the statistical test of interaction yields a p value of 0.06 (unpublished observation). Additionally, when the hazard ratio for Brazil (the only country where TcII is supposed to be most prevalent) is compared with all other countries combined, the p value for interaction is 0.03 (unpublished observation), indicating that the efficacy of benznidazole in Brazil was significantly different from the rest.

These findings show that such post-hoc analyses of geographical variations can be twisted to present either a more positive or a more negative impression of heterogeneity, depending on the comparisons made. As no grouping of countries is completely satisfactory, and subgroup analyses are generally seen as exploratory, a not unanimous decision by the BENEFIT steering committee was to show only the nonsignificant view of heterogeneity (p = 0.16) (Morillo et al. 2015).

Additionally, the results of the BENEFIT trial in Brazil should be analysed more judiciously (Table VI, Figure). Benznidazole reduced the relative risk of the primary endpoint by 15% (HR 0.85, 95% CI 0.71-1.02; p = 0.06) and the absolute by 4.4% compared with placebo. This reduction showed a strong trend toward statistical significance, and, had it reached significance, the number needed to treat to prevent the occurrence of one primary endpoint would be 22. We believe that this is a good result for an inexpensive drug that is given for a relatively short period of time (60 days) to patients with a chronic and often disabling disease, and has side-effects that are quite tolerable.

*Geographical variations in clinical outcomes in the BENEFIT trial* - Despite protocol-mandated uniform inclusion and exclusion criteria, some variation in the demographic characteristics of patient population and outcomes is usually expected in international randomised trials. However, in the BENEFIT trial, the difference in the proportion of patients in the placebo group who reached the primary endpoint was unusually large between geographical regions. The event rate (overall and per year) was lower than expected in patients from Argentina and Bolivia, i.e. it was approximately half the rate of those from Brazil (Table VI). Possible reasons for this difference are: patient selection; racial or genetic issues; regional disease characteristics; differences in background therapy, healthcare systems, and patterns of follow-up; and data quality issues, including protocol deviations.

The large subgroup of patients from Argentina and Bolivia (a third of the overall population), with event rates lower than anticipated, most likely diluted the effect size in the trial. Although large-scale international trials are preferable to those in one country, increasing recruitment by including other geographical regions may have obscured important treatment differences in the BENEFIT trial. Table VI suggests that if the study had been done in Brazil only, the results would probably have been positive.

*Conclusions and implications* - The hypothesis that trypanocidal treatment could benefit patients with established chronic Chagas cardiomyopathy was not confirmed by the results of the BENEFIT trial. Although the BENEFIT trial had been conceived originally as a pragmatic study, focusing on hard clinical endpoints, an unjustified emphasis on the results related to PCR-based detection of parasites in the blood led to the erroneous causal inference that despite enhancing circulating parasite clearance, the treatment with benznidazole did not result in clinical benefit for the patients with established chronic Chagas cardiomyopathy (Morillo et al. 2015). We caution against this biased interpretation, because of the erratic behavior of the PCR-based detection of the circulating parasite materials, and also because we do not have any information concerning parasite persistence in the myocardial tissue after treatment. Therefore, the BENEFIT trial results should not be understood as meaning that the main theory of Chagas cardiomyopathy pathogenesis (based on the parasite persistence), ought to be abolished. In fact, it is necessary to devise more effective treatment regimens and more effective methods to assess parasite clearance after aetiological therapy, if the hypothesis is to be tested again.

In the absence of more definitive data and with no other outcome trial planned for the near future, mainly due to the lack of interest from the pharmaceutical industry, doctors and patients with chronic Chagas cardiomyopathy must use the best available information to guide their treatment decision.

The overall neutral findings of the BENEFIT trial failing to show a significant reduction in the primary endpoint of morbidity and mortality in patients with chronic Chagas cardiomyopathy with the post-hoc observation that fewer patients receiving benznidazole are admitted to hospital for cardiovascular causes (Morillo et al. 2015) is probably the most reliable result of the trial. The regional differences in responses to benznidazole, with a signal of benefit in patients randomised from Brazil, may highly confound the interpretation of the results. Until more comprehensive data are available to better understand the differences in clinical outcomes between countries, the demographic characteristics of the patient population and their response to benznidazole, we expect a mixed response from the medical community. Some doctors will be reluctant to use benznidazole for patients with chronic Chagas cardiomyopathy, while others (including us) will continue to prescribe it, especially for most of our Brazilian patients.

We strongly believe that the risk of incurring an a error (not to apply a promising therapeutic intervention with tolerable side-effects) is much less acceptable than incurring a b error (not to adopt something that might prove futile in the future) (Marin-Neto et al. 2015b). On the basis of current evidence, doctors’ failure to even consider the possibility of aetiological treatment for their patients is questionable from an ethical standpoint (Tarleton et al. 2007, 2014, Viotti et al. 2014) – after all, “absence of evidence is not evidence of absence” (Altman & Bland 1995).

*Search strategy and selection criteria* - References for this Review were identified by searching PubMed and Google Scholar with the search terms “Chagas disease”, “Chagas heart disease”, “Chagas cardiomyopathy”, “American trypanosomiasis”, “*Trypanosoma cruzi*”, “pathology”, “pathogenesis”, “etiologic treatment”, “trypanocidal drugs”, “benznidazole”, “nifurtimox”, and “BENEFIT trial”. We reviewed relevant articles resulting from these searches, articles cited by those found, and articles present in our own files. We did not set any date or language limits.

**Figure:**
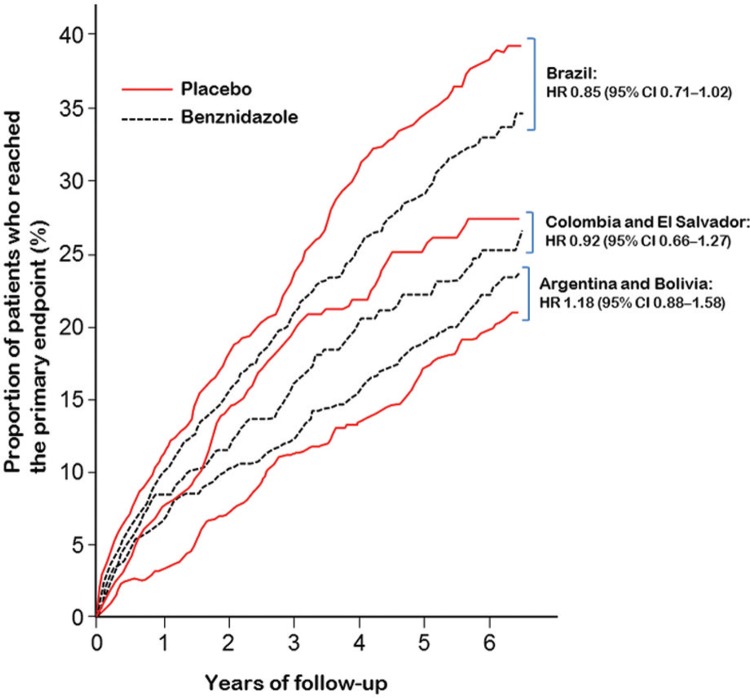
Geographical differences in the proportion of patients who reached the primary endpoint in the BENznidazole Evaluation for Interrupting Trypanosomiasis (BENEFIT) trial. HR = hazard ratio.
